# P-1227. Pulmonary Target Pharmacokinetic/Pharmacodynamic Attainment Rates among Cefepime-treated Patients Admitted to the ICU with Hospital-Acquired Pneumonia requiring Renal Replacement

**DOI:** 10.1093/ofid/ofaf695.1419

**Published:** 2026-01-11

**Authors:** Adrian Valadez, Emma Harlan, Marc H Scheetz, Helen K Donnelly, Erin Korth, Francisco J Martinez, Richard G Wunderink, Nathaniel J Rhodes

**Affiliations:** Midwestern University, Downers Grove, IL; Midwestern University, Downers Grove, IL; Midwestern University, Downers Grove, IL; Northwestern University, Chicago, Illinois; Northwestern University, Chicago, Illinois; Northwestern University, Chicago, Illinois; Northwestern University Feinberg School of Medicine, Chicago, IL; Midwestern University, Downers Grove, IL

## Abstract

**Background:**

The impact of continuous renal replacement therapy (CRRT) on cefepime (FEP) pharmacokinetics (PK) in the epithelial lining fluid (ELF) of patients with hospital-acquired pneumonia is not well described.

**Figure:**
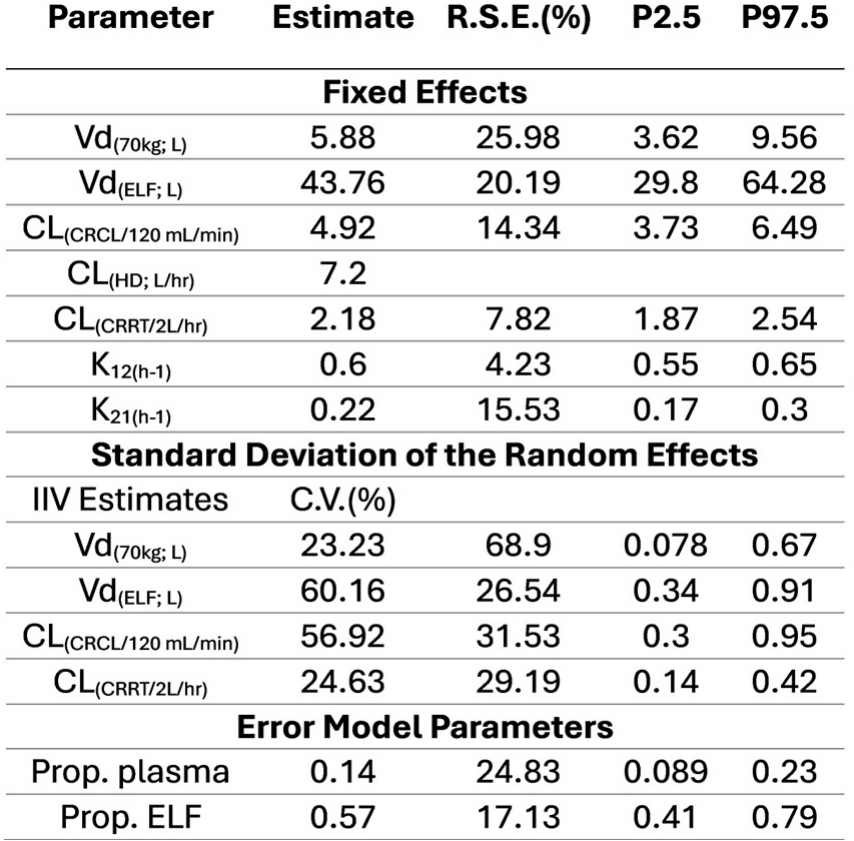
Population PK parameters for FEP in Renal Replacement in Plasma and ELF

**Figure:**
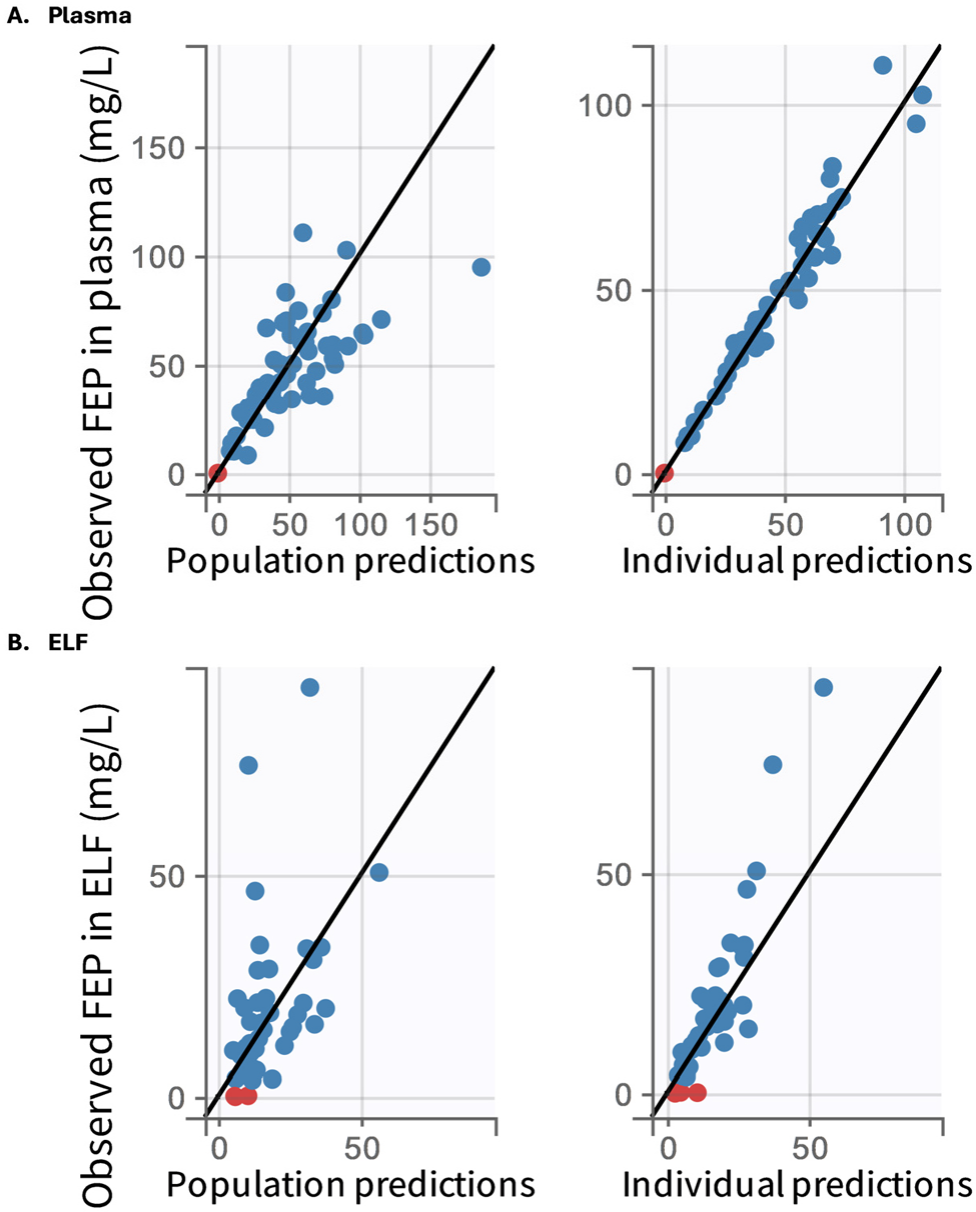
Observed vs. Predicted FEP in Plasma (A) and ELF (B) with BLQ samples(red)

**Methods:**

In this single center, nested within the Successful Clinical Response In Pneumonia Treatment (SCRIPT) study (June 2018 to March 2024), we evaluated FEP PK in adults with and without CRRT. Patient data were extracted from electronic health records. FEP dosing was according to institutional protocols based on CRRT flow and indication. FEP concentrations were quantified by validated LC-MS/MS assay. Population PK parameters [e.g., volume (V) and clearance (CL)] were estimated in Monolix 2024R1. Empirical Bayes estimates (EBEs) characterized individual PK profiles. In Simulx 2024R1, target attainment rates were calculated assuming a free (*f*) fraction of 80% vs. pharmacodynamic targets of 68% *f*T_> 1xMIC_ and 68% *f*T_> 4xMIC_ for *Pseudomonas aeruginosa (i.e., targets of* 8 and 32 mg/L), stratified by CRRT status (on/off circuit). Monte Carlo simulations (n=1000/regimen) compared attainment with 2 g every 12 hr as a 0.5 or 4 hr infusion without a loading dose.

**Figure:**
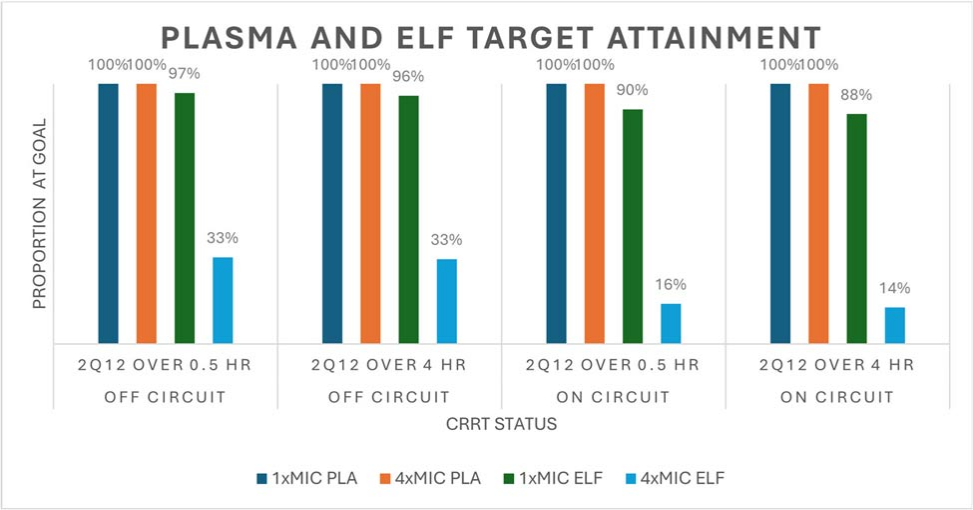
Probability of Target Attainment rates in Plasma and ELF Stratified by Circuit (on/off) Cefepime target-attainment rates for 68% fT>MIC at 1x and 4x MIC in epithelial lining fluid (ELF) and plasma for patients on versus off CRRT circuits (n = 1000 simulations).

**Results:**

Thirty-seven patients (63% male; mean weight 86.4 kg) contributed 56 plasma (1-3/patient) and 37 ELF (1-3/patient) samples. The mean±SD first 24-hr FEP dose was 4±2 grams. Mean on-circuit CRRT effluent flow was 3.2 L/hr; mean CRCL off circuit was 46 mL/min. A two-compartment PK model fit best (Fig 1), with body weight adjusted V and CRCL and effluent flow adjusted CL (Table 1). Patient-specific attainment was 100% for both *f*T _> 1xMIC_ and *f*T _> 4xMIC_ in plasma, which fell to 89% and 22% in ELF for *f*T _> 1xMIC_ and *f*T _> 4xMIC_ . Simulations using the population model are summarized in Fig 2. PLA attainment was high whereas ELF attainment at 1xMIC was >90% on but not off circuit with EI. Attainment of 4xMIC targets was achieved more often off circuit than on circuit (32-33% vs. 14-16% probability of attainment) .

**Conclusion:**

Standard cefepime dosing regimens achieved plasma PK/PD targets in CRRT patients but do not reliably reach high-level ELF exposures (4xMIC). CRRT patients on circuit had a lower probability of ELF attainment. Outcome evaluations in CRRT-dependent FEP-treated patients are warranted.

**Disclosures:**

Marc H. Scheetz, PharmD, MSc, Doseme: Advisor/Consultant|other: Additional not relevant to this abstract. If more information is needed about unrelated relationships, I can provide it. Nathaniel J. Rhodes, PharmD MS, Apothecademy, LLC: Advisor/Consultant

